# Influence of rigid taping on the acromiohumeral distance in healthy recreational weightlifters

**DOI:** 10.7717/peerj.12093

**Published:** 2021-08-26

**Authors:** Rodrigo Martín-San Agustín, Alba Cuerda-Del Pino, Noemi Moreno-Segura, Adrian Escriche-Escuder, Mariana Sánchez-Barbadora

**Affiliations:** 1 Department of Physiotherapy, Universidad de Valencia, Valencia, Spain; 2 Department of Physiotherapy, Malaga University, Malaga, Spain

**Keywords:** Acromiohumeral distance, Shoulder taping, Rigid taping, Sham taping, Ultrasound/Ultrasonography

## Abstract

**Background:**

Subacromial pain syndrome is one of the most frequent injuries in overhead athletes, and it takes place when the acromiohumeral distance (AHD) is narrowed. Conservative treatment is the first approach to this syndrome, being shoulder taping one of the most used techniques. Although there are quite a few studies that analyse the effect of taping on the AHD, most of them do not include sham tapings. This study aimed to examine if the Relocation of the humeral head (RHH) taping produced an increase in the AHD in healthy recreationally weightlifter males, quantifying the change that may be due to a placebo effect.

**Methods:**

The design of this study was a two-group pretest-posttest, in which eighteen healthy recreationally weightlifter males were measured. in a laboratory of the University of Valencia. RHH using rigid or sham taping was randomly applied to the participants. The AHD was measured and registered before and after the application of the taping for both groups by a blinded examiner using ultrasound.

**Results:**

There were no significant differences between pre and post measures in the sham group (*p* = 0.51). The experimental group showed a significant AHD increase of 9.2% (10.75 ± 1.89 *vs* 11.74 ± 1.82, respectively, with *p* < 0.001). Significant differences in the effects of each taping on the AHD were found between groups (*p* < 0.001). The results of this study indicate that the RHH rigid taping increases the AHD in the shoulders of recreationally weightlifters, dismissing the possibility of a placebo effect of the taping.

## Introduction

Proper shoulder kinematics are necessary to prevent the subacromial pain syndrome (most often referred to as shoulder impingement syndrome), one of the most frequent injuries in overhead athletes (Bigliani & Levine, 1997; Thelen, Dauber & Stoneman, 2008; Şimşek et al., 2013; Juel & Natvig, 2014). This syndrome takes place when the acromiohumeral distance (AHD), the distance between the upper part of the shoulder head and the acromion (Luque-Suarez et al., 2013), is narrowed as a result of damage of the bursa or the supraspinatus tendon (Lyman et al., 2017). Intrinsic and extrinsic mechanisms have been directly related to reducing this space, as well as the modified shoulder kinematics and the overactivation of the deltoid and supraspinatus (Solem-Bertoft, Thuomas & Westerberg, 1993; Michener, McClure & Karduna, 2003; Lyman et al., 2017).

Along with the medical history, clinical examination, and functional tests, the use of imaging techniques for the diagnosis of this pathology is widely used (Singh, Bakti & Gulihar, 2017). Ultrasound is a preferred tool over radiography or magnetic resonance for its quality of recording the image in motion and the preferred arm position (Azzoni, Cabitza & Parrini, 2004; Desmeules et al., 2004; Saupe et al., 2006).

Conservative treatment is the first approach to the subacromial pain syndrome, with shoulder taping as one of the most used techniques (Zanella et al., 2001; Cools et al., 2002; Selkowitz et al., 2007; Thelen, Dauber & Stoneman, 2008). A recent review has studied the relationship between the AHD and the existence of symptoms such as pain and disability, suggesting the importance of also addressing other biopsychosocial factors when dealing with a symptomatic patient (Park et al., 2020). There are many different studies where taping is applied to modify shoulder articular properties (*i.e.,* shoulder’s kinematics, muscle force, range of motion, neuromuscular control, pain or joint stability) (Host, 1995; Ackermann, Adams & Marshall, 2002; Lewis, Wright & Green, 2005; Thelen, Dauber & Stoneman, 2008; Shaheen et al., 2013; Harput et al., 2017), some of them also demonstrating the effectiveness of the taping to increase the AHD (Luque-Suarez et al., 2013; Lyman et al., 2017; Bdaiwi et al., 2017; Harput et al., 2017). However, some of these effects may be related to the placebo effect (Sawkins et al., 2007; Gulpinar, Tekeli Ozer & Yesilyaprak, 2019).

The relocation of the humeral head (RHH) taping is a technique specifically described to increase the space between the humeral head and the acromion, whose effect has not yet been studied (MacDonald, 2010). Previous to its application in injured population, it is important to know its effects in healthy people to quantify any possible physiological changes in the shoulder region independently of the pathology (Lyman et al., 2017). Thus, the aim of this study was to examine if the RHH taping produced an increase in the AHD in healthy recreationally weightlifter males, quantifying the change that may be due to a placebo effect.

## Materials & Methods

### Study design

**Figure 1:**
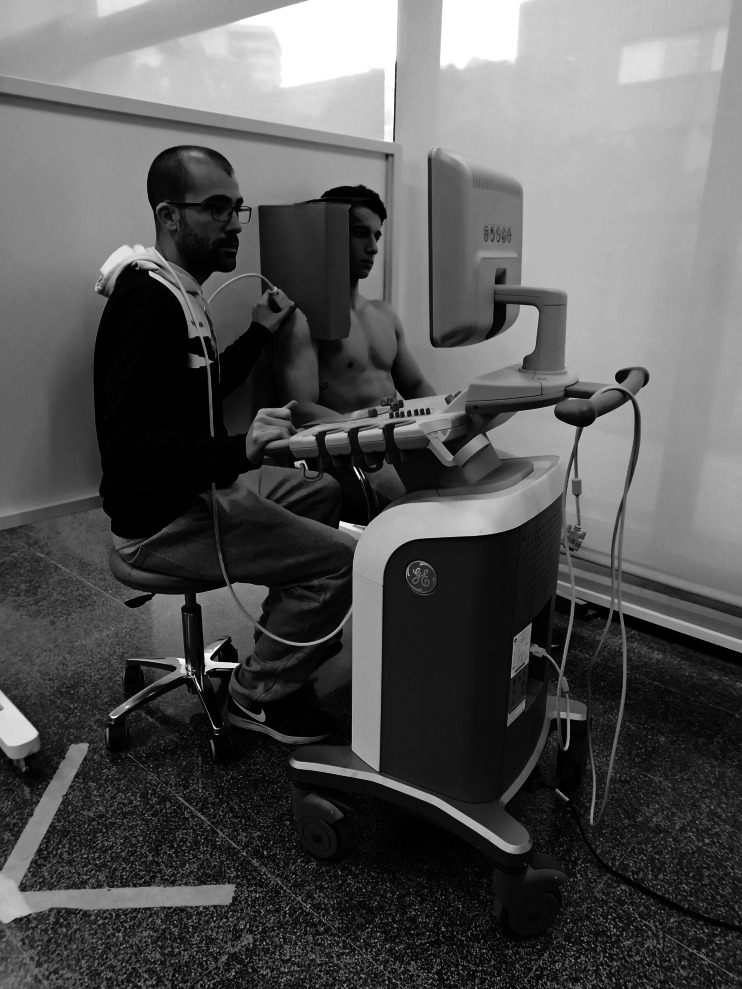
Ultrasound measurement. Photo credit: Rodrigo Martín-San Agustín.

A two-group pretest-posttest design was used to compare the effects of taping application on AHD in healthy participants, distributed in a sham group or an experimental group, using a randomised number system and a blinded assignment. AHD was measured before and after the intervention by one of the two examiners who carried out the study, blinded to the participant’s group. The second examiner applied the taping (sham taping or rigid taping). The examiner in charge of measuring the AHD left the room after the pre-intervention measurement had been made. Once the other examiner applied the taping, the participant occupied again the position seated on a chair leaving only the shoulder visible for ultrasound examination (as can be seen in Fig. 1). The first examiner then entered the room to perform the post-intervention measurement so that he could not know which taping was applied to each subject.

The physiotherapist in charge of doing the ultrasound exam had more than ten years of experience in musculoskeletal ultrasound. In the same way, the examiner who applied the taping was a physiotherapist with more than six years of experience in the sports field and had specific knowledge about taping.

Approval for the study was received from the Ethics Committee of the University of Valencia (Spain) (1169067).

### Subjects

Eighteen healthy recreationally weightlifter males were recruited by email using the University of Valencia Intranet by a professor from the same university. Each subject had at least 2 years of resistance training experience. For inclusion, participants needed to meet the following criteria: (I) be male, (II) be between the ages of 18 and 35 years, (III) have no history of shoulder surgery, (IV) have no history of shoulder pain in none of the shoulders two months previous to the participation, (V) have no history of other musculoskeletal injuries diagnosed by image and (VI) performing at least 120 min a week of strength training involving specific shoulder exercises overhead (*e.g.*, push press, push jerk, or split jerk). The only exclusion criterion was the contraindication of the use of the tape either by injury or adhesive allergy. All participants provided informed consent and completed an information sheet prior to data collection, including demographic (age, gender) and anthropometric measures (height, weight).

### Procedures

Ultrasound measurement (GE LOGIQ C5 Premium) of the AHD was performed in the dominant arm following the one described by J.S. Lewis (McCreesh et al., 2014). A customised opaque screen was used to blind the examiner, both in the pre-intervention AHD measurement and post-intervention AHD measurement. Participants were seated on a chair in a normal resting posture: shoulder in 0° of abduction, with the humerus hanging vertically along the side of the participant’s body, 90° elbow flexion and forearm pronation. The back supported, feet flat on the floor, hips and knees at 90° of flexion, head and shoulder in their habitual posture, looking straight ahead (Duerr, 2010; Kalra et al., 2010). The forearms and hands were placed on the armrests. The measurement was conducted from a superior imaging view, according to the methods described by Duerr (2010). The transducer was positioned horizontally on the anterior shoulder, visualising the landmarks of the coracoid process and the humerus. Maintaining the landmark of the coracoid process, the lateral part of the transducer was moved in a cranial direction until the anterior edge of the acromion was visualised. The coracoacromial ligament was identified between the bony landmarks of the coracoid process and the anterior edge of the acromion. Subsequently, the transducer was placed perpendicular to the axis of the coracoacromial ligament, producing an image of the AHD with the landmarks of the anterior edge of the acromion, the humeral head, and the greater tuberosity. Previously, the intra-rater reliability of this technique has shown an intraclass correlation coefficient (ICC) of 0.87 (0.81–0.92) and a minimal detectable change (MDC) of 0.9 mm (Duerr, 2010).

The shortest distance between the external inferior edge of the acromion and the most superior aspect of the surface of the humeral head was recorded, along the parallel line to the acoustic shadow emitted by the acromion. Three AHD measurements were made before and after applying the taping in both groups, waiting 20 min from the application of the taping for the post-taping measurement.

**Figure 2:**
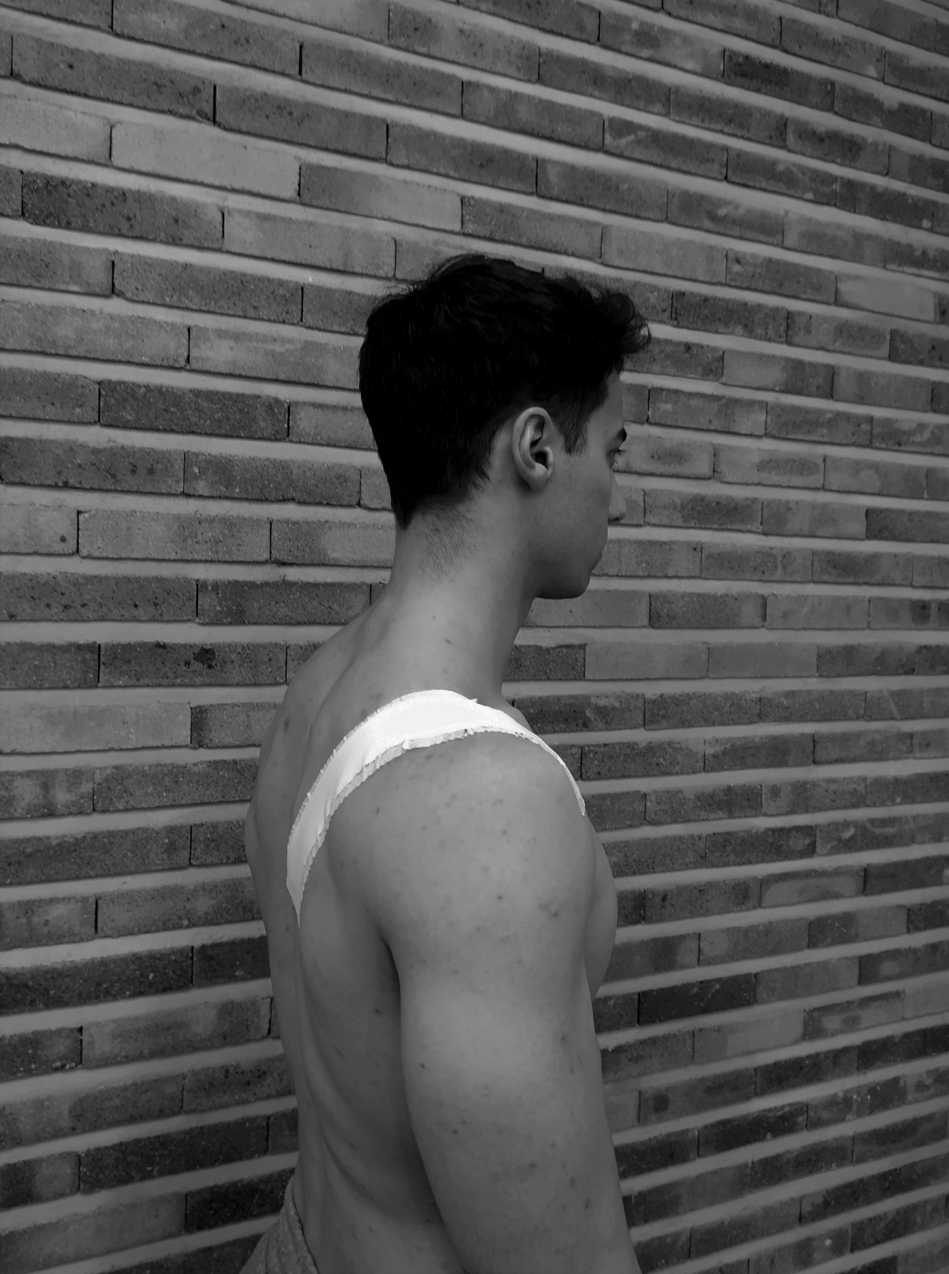
Relocation of the humeral head taping technique. Photo credit: Rodrigo Martín-San Agustín.

After baseline AHD measurement, the rigid tape was applied. The experimental taping used was the RHH, described by McConnell (MacDonald, 2010). This technique corrects the positional fault by lifting the anterior aspect of the humeral head up and back, increasing the space between the humeral head and the acromium. First, the Hypafix tape (BSN Medical) was placed on the area where afterwards the rigid tape would be settled. Then a strip of Strappal tape (BSN Medical) was anchored on the anterior aspect of the glenohumeral joint. With the thumb of the other hand, the humeral head was lifted up and back, pulling the tape stripe firmly diagonally across the scapula, and ending medial to the inferior border of the scapula. Hypafix has been used as a hypoallergenic tape, and the 3.8 cm Strappal tape has been used as a rigid tape (Fig. 2).

The sham taping used was applying the Hypafix tape, following the same procedure as in the experimental group but placing it on the skin without any tension and without the Strappal tape stripe.

### Statistical analysis

Participants’ characteristics were summarised as means (SD) and AHD measurements as means (95% confidence intervals (CIs)). The normality of distribution for the AHD was evaluated by Shapiro–Wilk test. However, in light of the sample size of this study, normality tests for AHD were performed from the pre–post differential value (Martín-San Agustín et al., 2020). Unpaired *t*-tests were used to examine the differences in demographic, anthropometric characteristics, and AHD at baseline between groups. Paired *t*-tests were used in each group to analyse the differences between the AHD before and after each taping. Cohen’s *d* was also calculated to evaluate the effect size (*d* < 0.2: trivial, 0.2–0.5: small, 0.5–0.8: medium, and >0.8: large) (Cohen, 2013). Cohen’s *d* > 0.5 was considered to be a practically important difference. Finally, unpaired *t*-tests were used to detect between-group differences in AHD, using pre–post differential value as dependent variable. The effect size was evaluated with *η*^2^ (Eta partial squared) (Morris, 2008; Lenhard & Lenhard, 2017) where 0.01< *η*^2^ < 0.06 constitutes a small effect, a medium effect when 0.06<*η*^2^<0.14, and a large effect when *η*^2^ > 0.14 (Cohen, 2013).

A sample size of 9 participants in each group was calculated to provide sufficient statistical power (80%) to detect an effect size of 1.28 for the difference in AHD changes between groups (Luque-Suarez et al., 2013), with alpha level of 0.05.

## Results

Table 1 shows the participants’ characteristics. The mean ages of the sham and the experimental groups were 24.0 (SD = 5.7) and 23.2 (SD = 2.9), respectively. The mean BMI were 23.9 kg/m^2^ (SD = 2.1) for the sham group and 22.7 kg/m^2^ (SD =2.7). Both groups showed a weekly physical activity superior to 326 min [(sham group = 326.7 min (SD = 177.8) and experimental group = 396.6 min (SD = 205.5)]. No differences between groups were shown for any characteristic.

**Table 1:** Demographic and anthropometric characteristics of the participants (*n* = 18). Mean values (SD) except to laterality.

	Sham group (*n* = 9)	Experimental group (*n* = 9)
Age (years)	24.0 (5.7)	23.2 (2.9)
Body mass (kg)	69.7 (9.1)	78.0 (9.2)
Stature (cm)	175.2 (6.5)	180.2 (7.6)
BMI (kg/m2)	23.9 (2.1)	22.7 (2.7)
Weekly physical activity (minutes)	326.7 (177.8)	396.6 (205.5)
Laterality	Right (*n* = 7)	Right (*n* = 7)

There were no significant differences between pre and post measures in the sham group (mean difference = −0.07 mm; *d* =  − 0.04). However, the experimental group showed a significant AHD increase of 9.2% (mean difference = 0.99 mm; *d* = 0.53). Significant differences were found between the effects of each taping on the AHD (*p* < 0.001) (Table 2), associated with a large effect size (*η*^2^ =0.79). Differences between groups in the pre-post changes in AHD were 1.06 mm.

**Table 2:** Means and standard deviations for the measurement of the AHD^a^. Differences between pretest and posttest measurements and between interventions.

	Mean AHD (95% CI)			Sham *vs* experimental
	Pretest	Posttest	Mean difference (95% CI); *p* value	*p* value; effect size (*η*^2^)
Sham group	10.84 (9.60–12.09)	10.77 (9.60–11.95)	−0.07 (−0.30-0.16); 0.509	0.001; 0.79
Experimental group	10.75 (9.29–12.21)	11.74 (10.34–13.14)	0.99 (0.80-1.18); 0.001	

**Notes.**

AHD acromiohumeral distance SD standard deviation CI confidence interval ES effect size

a AHD expressed in mm.

## Discussion

In this study, we analysed the changes in the AHD when two different tapings were applied, one rigid and one sham, using ultrasound to measure the AHD before and after its application. The main finding was the increase of the AHD when the RHH taping was applied. Furthermore, we proved that the difference was independent of a possible placebo effect.

According to our knowledge, this is the first study that compares a rigid taping with a sham taping to increase the AHD. In a previous study, the efficacy of two rigid tapings, in isolation or combined, was demonstrated to increase the AHD, obtaining similar results to ours (Bdaiwi et al., 2017). However, they have not included a sham taping to compare their technique. As mentioned before, previous studies have found that some of the effects of taping could be related to a placebo effect (Sawkins et al., 2007; Gulpinar, Tekeli Ozer & Yesilyaprak, 2019), so this limitation in their methodology may make the taping used by us a better option to obtain results of increasing the AHD not conditioned by the placebo effect.

Other studies have analysed the effects of different elastic tapings on the AHD in healthy subjects, placing the tape stripes in different directions and positions (Luque-Suarez et al., 2013; Lyman et al., 2017; Harput et al., 2017). These studies obtained similar results to the present work, with gains in the distance of around 7–12%. Moreover, one of them also included a comparison with a sham elastic taping , setting aside the placebo effect (Luque-Suarez et al., 2013). Thus, it seems that our rigid taping compared to other elastic tapings could have similar effects increasing the AHD, making the use of either of them appropriate in clinical practice.

The RHH is a technique indicated in cases of anterior shoulder instability, impingement problems, rotator cuff tears and adhesive capsulitis (MacDonald, 2010). The physiological mechanism by which taping works is not fully understood. Several authors have speculated on this and have seen that the taping increases muscle force (Host, 1995). It is also possible that rigid taping passively repositions the scapula and hence influence the AHD (Bdaiwi et al., 2017). In any case, more research would be necessary on the physiological changes produced by the application of taping.

As previously mentioned, treatment of a patient with subacromial pain syndrome should not only focus on addressing a possible decrease in the AHD, but also on the importance of other biopsychosocial factors (Park et al., 2020). Nevertheless, according to the authors the evidence is still limited to determine the correlation between AHD and pain or disability, as the included studies had different outcome measures and different study population (Park et al., 2020). The differences in the AHD found in our study justify the use of this technique to obtain an increase in the distance in healthy subjects. When it comes to injured population, other approaches besides taping would be indicated.

The main strength of our study was the methodology used, the randomised assignment of groups, and the blinding of the examiners. Moreover, the inclusion of the sham group rejects the possible placebo effect of the technique. Furthermore, the differences between groups for the AHD (1.06 mm) exceed the smallest real difference (*i.e.,* the smallest measurement change that can be interpreted as a real difference) of the measurement technique used, being able to consider that the changes obtained are independent of the measurement error.

However, although the statistically significant obtained results are encouraging, this study also has several limitations. First, the effects are measured only in the short term, so we can not guarantee the efficacy of the technique longer in time. In addition, the study was carried out including only healthy recreationally weightlifter males, so more investigation in females, injured and, athletes of other overhead sports (*e.g.*, volleyball or basketball) is necessary to extrapolate these results to the clinical practice.

## Conclusion

The results of this study indicate that the RHH taping increases the AHD in shoulders of recreationally weightlifter males. Furthermore, the application of a sham taping did not produce any change in the AHD

##  Supplemental Information

10.7717/peerj.12093/supp-1 Supplemental Information 1 Raw measurements

10.7717/peerj.12093/supp-2 Supplemental Information 2 Codebook

## Additional Information and Declarations

### Data Availability

The following information was supplied regarding data availability: The raw measurements and the codebook are available in the Supplementary Files.
