# Supplementary material for: Influence of rigid taping on the acromiohumeral distance in healthy recreational weightlifters
Source: PeerJ. 2021 Aug 26;9:e12093. doi: 10.7717/peerj.12093 (PMC8403474; doi:10.7717/peerj.12093)
Supplement: Supplemental Information 2 [file peerj-09-12093-s002.docx]

Variable group:

1- Experimental

2-Sham
